# Transcriptomics and Other Omics Approaches to Investigate Effects of Xenobiotics on the Placenta

**DOI:** 10.3389/fcell.2021.723656

**Published:** 2021-09-24

**Authors:** Cheryl S. Rosenfeld

**Affiliations:** ^1^Biomedical Sciences, University of Missouri, Columbia, MO, United States; ^2^MU Institute for Data Science and Informatics, University of Missouri, Columbia, MO, United States; ^3^Thompson Center for Autism and Neurobehavioral Disorders, University of Missouri, Columbia, MO, United States; ^4^Genetics Area Program, University of Missouri, Columbia, MO, United States

**Keywords:** trophoblast, serotonin, bisphenol A, endocrine disruptors, environmental chemicals, placenta-brain axis, pharmaceutical agents, smoking

## Abstract

The conceptus is most vulnerable to developmental perturbation during its early stages when the events that create functional organ systems are being launched. As the placenta is in direct contact with maternal tissues, it readily encounters any xenobiotics in her bloodstream. Besides serving as a conduit for solutes and waste, the placenta possesses a tightly regulated endocrine system that is, of itself, vulnerable to pharmaceutical agents, endocrine disrupting chemicals (EDCs), and other environmental toxicants. To determine whether extrinsic factors affect placental function, transcriptomics and other omics approaches have become more widely used. In casting a wide net with such approaches, they have provided mechanistic insights into placental physiological and pathological responses and how placental responses may impact the fetus, especially the developing brain through the placenta-brain axis. This review will discuss how such omics technologies have been utilized to understand effects of EDCs, including the widely prevalent plasticizers bisphenol A (BPA), bisphenol S (BPS), and phthalates, other environmental toxicants, pharmaceutical agents, maternal smoking, and air pollution on placental gene expression, DNA methylation, and metabolomic profiles. It is also increasingly becoming clear that miRNA (miR) are important epigenetic regulators of placental function. Thus, the evidence to date that xenobiotics affect placental miR expression patterns will also be explored. Such omics approaches with mouse and human placenta will assuredly provide key biomarkers that may be used as barometers of exposure and can be targeted by early mitigation approaches to prevent later diseases, in particular neurobehavioral disorders, originating due to placental dysfunction.

## Introduction

The placenta and uterine tissue directly interact, and thus, factors circulating in the maternal bloodstream easily transfer across the placenta, where they can affect this organ and secondarily the fetus. This close relationship between the placenta and maternal tissue is essential for nutrient, gas, and waste exchange. The placenta is also an endocrine organ that produces a range of hormones and cytokine factors that exert local paracrine effects in the placenta but can also act upon maternal and fetal tissues. Many biomedical studies examine effects on mouse or rat placenta as rodents have an invasive hemochorial type of placentation with syncytiotrophoblast (syncytioTB) cells that are involved in nutrient and gas exchange and are bathed in maternal blood, analogous to structural components of the human placenta (Rosenfeld, 2015a). However, the fetal placental cells, trophoblasts (TB), may also be immersed in compounds percolating through the maternal blood. For this reason, TB cells have some ability to detoxify select xenobiotic chemicals, which may help buffer the fetus against such chemical assaults (Myllynen et al., 2005; Obolenskaya et al., 2010; Corbel et al., 2015; Nahar et al., 2015). However, being an endocrine organ in of itself, the placenta is vulnerable to a myriad of exogenous chemicals. The ability to respond to such environmental challenges is also likely sexually dimorphic in nature (Mao et al., 2010; Rosenfeld, 2015a).

Individual gene or protein expression patterns were used to ascertain the effects of such chemical exposures on the placenta. Microarray technology was the first method employed to relate such chemicals exposures and transcriptomic changes in the placenta (e.g., Imanishi et al., 2003; Avissar-Whiting et al., 2010; Bruchova et al., 2010; Votavova et al., 2011; Tait et al., 2015; Grindler et al., 2018). However, such studies were confined to genes included on the arrays, and microarrays were only developed for a few select species whose genome was sequenced and annotated. RNA sequencing (RNAseq) is a high throughput approach that has greatly expanded our knowledge of how EDC, other environmental toxicants and pharmaceutical agents affect global gene expression patterns in the placenta (e.g., Green et al., 2020; Mao et al., 2020). Herein, we will consider the studies to date that have shown such extrinsic factors can affect transcriptomic profiles or protein expression in the placenta as determined by microarray analyses, RNAseq, or candidate gene/protein approaches. Further, we will consider other omics approaches, including metabolomics, proteomics, and methylomics, that have been used to characterize the effects of xenobiotics on the human and rodent placenta. The importance of miRNAs (miRs) is gaining currency as such small RNAs have the ability to block translation by binding to target mRNA (Van Wynsberghe et al., 2011; Moreno-Moya et al., 2014). The miR/mRNA complexes can then be degraded prior to the mRNA entering the cytoplasm to be translated into a protein. In this way, miR represent the final epigenetic regulators. Studies have thus examined how some of the above factors can regulate miR and other non-coding RNA profiles by using small RNAseq. Lastly, we will consider some of the recently developed transcriptomic technology that will continue to advance our understanding in placental toxicology and allow for pinpointing transcriptomic changes in individual TB cell populations.

### Endocrine Disrupting Chemicals

Endocrine disrupting chemicals (EDC) are synthetic and natural compounds that can mimic or antagonize endogenous hormone responses. BPA is a widely prevalent synthetic chemical that can act through steroid and non-steroid receptor pathways (Schug et al., 2011). Current production estimates for BPA are around 20 billion pounds (Grand View Research, 2014). Approximately 93% of the U.S. population unknowingly has measurable amounts of BPA in their urine (Calafat et al., 2008). Exposure to BPA and, its analog, bisphenol S (BPS) is primarily dietary (Galloway et al., 2010; Sieli et al., 2011), but other routes of exposure are known (Xue et al., 2016; Hines et al., 2018). BPA readily can be transmitted from the maternal tissue to the fetal placenta (Vandenberg et al., 2007; vom Saal et al., 2007). BPA substitutes, such as BPS, are increasingly being used in a range of consumer products labeled BPA-free. Yet, BPS may lead to similar and potentially even more pronounced effects compared to BPA (Rosenfeld, 2017; Wu et al., 2018).

BPA has been shown to affect placental gene expression patterns (Imanishi et al., 2003; Kang et al., 2011; Susiarjo et al., 2013; Tan et al., 2013; Tait et al., 2015; Xu et al., 2015; Lee et al., 2016; Lan et al., 2017). Most of these reports though only used a candidate gene expression approach (Kang et al., 2011; Susiarjo et al., 2013; Tan et al., 2013; Tait et al., 2015; Xu et al., 2015; Lee et al., 2016; Lan et al., 2017), in particular for those known to be imprinted (Kang et al., 2011; Susiarjo et al., 2013). More recent studies employed microarrays to examine thousands of genes in a single experiment (Imanishi et al., 2003; Tait et al., 2015), but such studies may have been under-powered. One study that used microarrays to examine the effects of BPA on the placenta found that the high dosage of BPA tested resulted in significant degeneration and necrosis of giant cells, vacuolization in the junctional zone, and overall reduction of the spongioTB layer (Tait et al., 2015). Nuclear accumulation of β-catenin was evident in TB within the labyrinthine and spongioTB layers, suggestive of Wnt/β-catenin pathway activation (Tait et al., 2015). The microarray studies revealed that the low dosage of BPA tested promoted blood vessel development and arborization, whereas the high dose inhibited such angiogenic changes.

We used RNAseq analyses to examine the global transcriptomic profile in embryonic age (E) 12.5 mouse placenta following dietary exposure to BPA or BPS. BPA and BPS altered the expression of an identical set of 13 genes (Mao et al., 2020). Of which, 11 were downregulated and two (*Actn2* and *Efcab2*) modestly upregulated. Four of the differentially expressed (DE) transcripts are typically enriched in the placenta (*Sfrp4*, *Coch*, *Gm9513*, and *Calm4*) as determined by the TissueEnrich program (Mao et al., 2020). Based on the DE gene-sets, WNT and chemokine signaling pathways, amino acid metabolism, and possibly neurotransmission are pathways predicted to be affected in the placental samples exposed to BPA/BPS.

In the same study, we examined for histopathological changes in the placenta following BPA and BPS exposure. Additionally, targeted and non-targeted metabolomics analyses were performed to determine the extent to which these EDCs affect other omics profiles and whether transcriptomics and metabolomic changes correlated with BPA/BPS-associated architectural modifications in the placenta (Mao et al., 2020). Both exposures reduced the area occupied by spongioTB relative to parietal trophoblast giant cells (pTGC) within the junctional zone. Both BPA and BPS markedly reduced placental serotonin (5-HT) concentrations and lowered 5-HT pTGC immunoreactivity. Concentrations of dopamine and 5-hydroxyindoleacetic acid), the main metabolite of 5-HT, however, were increased. Dopamine-immunoreactivity in pTGC was increased in BPA and BPS exposed placentas. By using mixOmics analyses (Rohart et al., 2017), we found a strong positive correlation between 5-HT positive pTGC cells and reductions in spongioTB to pTGC area, indicative that 5-HT is essential for maintaining cells within the junctional zone. In contrast, an inverse correlation existed between dopamine positive pTGC cells and reductions spongioTB to pTGC area. The collective findings suggest that BPS exposure causes almost identical placental effects as BPA. A major target of BPA/BPS is either spongioTB or pTGC within the junctional zone. BPA/BPS induced disruptions in placental 5-HT and dopamine may affect fetal brain development through the placenta- brain axis. It is clear that 5-HT as a morphogen may be one of the primary conductors regulating early neural crest formation, metamorphosis, neurogenesis, cell motility, synaptogenesis, and development of the nociceptive system (Lauder, 1988; Lauder et al., 1988; Whitaker-Azmitia, 1991; Herlenius and Lagercrantz, 2001). Strong evidence exists that the initial source of 5-HT to orchestrate such neural changes is the placenta (Huang et al., 1998; Bonnin and Levitt, 2011; Hadden et al., 2017). By using such omics approaches in the placenta, it may also thus shed light on how EDC compromise early neural development and thereby increase the risk for neurobehavioral disorders.

BPA might also affect DNA methylation patterns in the placenta. One study examined BPA concentrations, gene expression patterns of BPA-specific metabolizing enzymes, and global DNA methylations in the placenta of 2nd trimester human fetuses (Nahar et al., 2015). Average LINE1 and CCGG global methylation in the placenta were 58.3 and 59.2%, respectively, Total BPA concentrations positively correlated with global methylation for the placenta based on the LINE1 assay. BPA-specific metabolizing enzymes, such as GUSB, UGT2B15, STS, and SULT1A1 were identified in these placenta samples. The findings suggest that maternal exposure to BPA might promote hypermethylation of select genes in the placenta.

Phthalates are another class of EDCs found in commonly used household items, including children’s toys, plastic containers, and plastic wraps (Ferguson et al., 2011; Lioy et al., 2015; Schulz et al., 2015; Jo et al., 2018). They are associated with adverse pregnancy outcomes, including fetal loss and placental growth abnormalities (Zong et al., 2015; Gao et al., 2017; Mahaboob Basha and Radha, 2017), but the full range of mechanisms by which they induce such effects remains elusive. Examination of the DNA methylome (Illumina Infinium Human Methylation 850k BeadChip) and transcriptome (Agilent whole human genome array) in first-trimester human placenta revealed 39 genes that demonstrated altered methylation and gene expression patterns in women exposed to high amounts of phthalates with most showing reduced expression in this group (Grindler et al., 2018). The combined usage of methylomics and transcriptomics revealed epidermal growth factor receptor (EGFR) as a likely primary mediator of phthalates on placental function.

Another cohort study revealed that chorionic gonadotropin A (*CGA*) showed sex-dependent gene expression changes in the placenta that were linked to various phthalate concentrations detected in the urine of pregnant women (Adibi et al., 2017a). *CY19A1*, *CYP11A1*, *CGA* expression in the placenta correlated with maternal urinary concentrations of monobenzyl phthalate (MBzP), MnBP, mono-iso-butyl phthalate (MiBP), and conceptus sex (Adibi et al., 2017a).

*In vitro* culture approaches have aided our understanding of how EDCs affect placental cells. One study used TB stem cells from rhesus monkeys (*Macaca mulatta*) to screen global transcriptome changes induced by several EDCs, atrazine, tributyltin, bisphenol A, bis(2-ethylhexyl) phthalate, and perfluorooctanoic acid (PFOA) (Midic et al., 2018). Atrazine and tributylin, and to a lesser extent the other three EDCs, suppressed genes involved in cytokine signaling related to antiviral response, along with those involved in metabolism, DNA repair, and cell migration.

Another study isolated human TB progenitor cells at 7–14 weeks. of pregnancy from two female and three male concepti, as well as villous cytotTB cells (vCTBs) at 15–20 weeks. pregnancy from three female and four male concepti. Primary cell lines were cultured in the presence of one or more phthalates: mono-*n*-butyl (MnBP), monobenzyl (MBzP), mono-2-ethylhexyl (MEHP), and monoethyl (MEP) (Adibi et al., 2017b). Treatment of both TB lines with MnBP, MBzP and MEHP at concentrations that resemble those found in the urine of pregnant women altered *CGB* and *PPARG* expression in these primary placental cells, although the effects varied according to the sex from which the placental cells were derived (Adibi et al., 2017b).

### Other Environmental Toxicants

Other environmental toxicants, including heavy metals, such as arsenic, and flame retardants can reach the placenta, whereupon they may induce transcriptomic changes. The earth’s crust contains arsenic, and it can also be found in the water, land, and air. However, it is highly toxic in the inorganic form (WHO, 2018). Common routes of exposure to inorganic arsenic are through drinking contaminated groundwater, using such water in food preparation and irrigation of food crops, manufacturing of it, consumption of contaminated food, and smoking tobacco (WHO, 2018). As with the EDCs, the placenta can accumulate high concentrations of arsenic that can lead to placental alterations, including in the glucocorticoid receptor pathway, oxidative stress, inflammation, linkages to pre-eclampsia, and epigenetic changes (Ahmed et al., 2011; Caldwell et al., 2015; Cardenas et al., 2015; Li et al., 2015; Green et al., 2016; Konkel, 2016; Appleton et al., 2017; Rahman et al., 2018; Punshon et al., 2019; Winterbottom et al., 2019b; Meakin et al., 2020; Stone et al., 2021).

To examine whether exposure to arsenic results in global transcriptomic changes in the placenta, 46 pregnant women were selected from the New Hampshire Birth Cohort Study (NHBCS), which is a US cohort known to have low-to-moderate arsenic levels in drinking water because of unregulated private wells (Winterbottom et al., 2019a). Potential sex-dependent gene expression changes in the placenta were correlated with prenatal exposure to arsenic, as determined by concentrations in the urine of pregnant mothers. While no genes were differentially expressed in female placenta based on arsenic exposure, several hundred genes were affected in the placenta of males. Two of the genes that showed the greatest downregulation in male placenta exposed to arsenic were *FIBIN* and *RANBP3L* (Ahmed et al., 2011; Caldwell et al., 2015; Cardenas et al., 2015; Li et al., 2015; Green et al., 2016; Konkel, 2016; Appleton et al., 2017; Rahman et al., 2018; Punshon et al., 2019; Winterbottom et al., 2019b; Meakin et al., 2020; Stone et al., 2021).

To understand how such gene expression patterns might originate in the placenta, a handful of studies have examined the expression of epigenetic regulator genes and DNA methylation profiles in the placenta of arsenic-exposed human cohorts. One study analyzed the expression of over a hundred epigenetic regulator genes, such as those that act as readers, writers and erasers of post-translational histone modifications, and chromatin remodelers (Winterbottom et al., 2019b). Several of these genes demonstrated differences based on the interaction between placental sex and arsenic exposure with the histone methyltransferase (*PRDM6*) negatively correlating with arsenic exposure. Placental glucocorticoid receptor (*NR3C1*) methylation positively associated with arsenic exposure (Appleton et al., 2017).

Global DNA methylation patterns based on CpG loci were examined in placental samples obtained from 343 individuals enrolled in the New Hampshire Birth Cohort Study and correlated based on arsenic levels in the urine and toenails samples of these pregnant mothers (Green et al., 2016). While no linkages were found based on arsenic in maternal urine, strong association were identified based on levels of this heavy metal in the toenail samples. Of the 163 differentially methylated loci, the primary one was for *LYRM2* (Green et al., 2016). This study also found that allocation of placental cell sub-populations changed based on arsenic exposure.

Another study linked arsenic exposure and DNA methylation patterns, as determined by Infinium HumanMethylation450 BeadChip array, in the placenta, in the umbilical artery, and human umbilical vein endothelial cells (HUVEC) (Cardenas et al., 2015). Genes regulating melanogenesis and insulin signaling pathways were differentially methylated in the placenta and umbilical artery based on arsenic exposure (Cardenas et al., 2015).

The flame-retardant mixture, Firemaster 550 (FM 550) contains organophosphate flame retardants that was hypothesized to disrupt placental function. To examine how this environmental toxicant affected the placenta, pregnant Wistar rats were treated with varying concentrations of this chemical (Rock et al., 2020). This treatment altered the expression of genes involved in transport and synthesis of 5-HT in the placenta (Rock et al., 2020). Additionally, metabolites of 5-HT and the kynurenine metabolic pathway were increased.

The cellular and transcriptomic effects of another flame retardant, BDE-47- a polybrominated diphenyl ethers (PBDEs), was tested in human placental cytotTB cells (CTBs) (Robinson et al., 2019). This compound suppressed migration and invasion by CTBs. BDE-47 induced transcriptome changes that were dose dependent with genes involved in stress, inflammation, lipid/cholesterol metabolism, differentiation, migration, and vascular morphogenesis affected (Robinson et al., 2019). Hypermethylation of CpG islands for genes involved in cell adhesion and migration occurred in response to this treatment.

### Pharmaceutical Agents

Pregnant women are often prescribed pharmaceutical agents to regulate such conditions as depression, epilepsy, and pain. While such drugs may be beneficial to the mother, they can have untoward consequences on the conceptus, including the placenta. Transcriptomics and other gene expression approaches have been useful tools in understanding how such xenobiotics alter the genetic machinery in this organ. In this section, we will consider three such pharmaceutical agents, serotonin-reuptake inhibitors (SSRI) used to treat depression, valproic acid (VPA) used to treat seizures, and oxycodone (OXY) that is a commonly prescribed analgesic agent.

Approximately 8–10% of pregnant women are prescribed selective serotonin-reuptake inhibitors (SSRI) to combat depression (Mitchell et al., 2011; Huybrechts et al., 2013). Such drugs act by binding to SLC6A4/SERT within the intracellular membrane, which in the central nervous system prevents the presynaptic cells from accruing 5-HT. Inhibition of SLC6A4/SERT results in increased concentrations of 5-HT in the synaptic space that can continue to bind and activates its cognate receptors. Such drugs though can also inhibit SLC6A4/SERT within placental TB, namely the pTGC that use this transporter to uptake maternal 5-HT. *In vitro* studies reveal SSRIs disrupt various structural and hormonal properties of placental cell lines (Hudon Thibeault et al., 2017; Clabault et al., 2018a,b). In rats, *in utero* exposure to venlafaxine reduced fetal placental weight (Laurent et al., 2016). Two SSRI, fluoxetine and sertraline, reduced cell proliferation of extravillous TB (JEG-3) cells (Clabault et al., 2018a). Norfluoxetine, a metabolite of fluoxetine, increased MMP-9 activity by these TB cells but suppressed MMP-9 activity in another cell line, HIPEC, derived from extravillous TB. TIMP-1 and ADAM-10 showed increased expression in JEG-3 cells treated with sertraline. Venlafaxine, another SSRI, increased ADAM-10 in HIPEC cells (Clabault et al., 2018a). Sertraline and venlafaxine induced fusion of cultured primary villous TB cells (Clabault et al., 2018b). Both compounds affect human chorionic gonadotropin beta (β-HCG) secretion by BeWo TB-derived cells. Norfluoxetine stimulated increased gene expression of chorionic gonadotropin beta (*CGB*) and gap junction protein alpha 1 (*GJA1*) which are considered biomarkers of syncytialization for these TB cells. Pregnant mothers consuming SSRIs have been reported to deliver lower birthweight infants and exhibit higher rates of placental-fetal vascular malperfusion than controls (Levy et al., 2020).

Valproic acid (VPA) is a short-chain-fatty acid commonly used as an antiepileptic drug and mood stabilizer (Chateauvieux et al., 2010). Such beneficial effects are ascribed to its inhibition of gamma amino butyric acid (GABA), transaminobutyrate, and ion channels (Chateauvieux et al., 2010). More recently, it has been shown that VPA can act as an histone deacetylase (HDAC) inhibitor that increases transcription by preventing deacetylation of histone proteins (Monti et al., 2009). Thus, some of the actions of VPA may be due to its epigenetic properties. Epilepsy is the most common neurological disorder in pregnant women, necessitating continued usage of antiepileptic drugs (AED) to prevent seizures. VPA is one of the primary AED prescribed to pregnant women, even though current data suggests that it may be associated with adverse fetal outcomes and behavioral deficits in children exposed *in utero* to this drug (Eadie, 2014; Elkjaer et al., 2018; Richards et al., 2019; Vajda et al., 2019; Daugaard et al., 2020). The placenta is also not immune to the effects of this AED (Khera, 1992; Meir et al., 2016; Tetro et al., 2019; Jinno et al., 2020; Shafique and Winn, 2021), and VPA-induced changes in the placenta may adversely affect fetal development, including the brain.

To examine the transcriptome changes in response to VPA, term placenta from women who delivered via cesarean were perfused with varying concentrations of VPA or vehicle. They were than analyzed with a customized gene array panel to examine the expression of carrier genes (Rubinchik-Stern et al., 2018). This drug treatment changed the mRNA expression patterns for transporters of folic acid, glucose, choline, thyroid hormone, and serotonin. Placental folate concentrations were also decreased with VPA treatment. VPA treatment to pregnant rats altered the expression of other transporter genes with *Abcc4* and *Slc22a4* reduced in late gestation, but *Abcc5* was increased by VPA during mid-gestation (Jinno et al., 2020). Whether such changes on transporter gene expression patterns in the human and rat placenta is due to HDAC inhibition or other biological effects of VPA remains uncertain.

Opioid drugs, especially oxycodone (OxyContin, OXY), are widely prescribed analgesic agents to control pain in pregnant women. This abuse is one of the leading non-infectious disease public health concerns and economic challenges facing the United States (Reinhart et al., 2018). Opioid use disorder (OUD), is a particular health concern in women of child-bearing age (SAMHSA, 2015) with OUD during pregnancy estimated to affect 5.6 per 1000 live birth infants (Patrick et al., 2012). Neonates exposed during gestation to opioids are at risk for neonatal abstinence syndrome (NAS) (Jones et al., 2019). Maternal OUD has been associated with poor fetal growth, increased risk for premature births, low birthweight offspring, and congenital defects (Yazdy et al., 2015; CDC, 2019). Adult-onset diseases due to developmental origin of health and disease (DOHaD) effects of these drugs are also possible (Grandjean et al., 2015; Rosenfeld, 2015b). The placenta may be bathed and affected by any opioids circulating in the maternal blood, whereupon it can affect this organ and be transmitted to the fetus.

An endogenous opioid system is present in the placenta that mediates several placental responses, including production of maternal recognition of pregnancy factors, such as HCG and placental lactogens (Cemerikic et al., 1991; Ahmed et al., 1992; Cemerikic et al., 1992; Petit et al., 1993; Cemerikic et al., 1994). Exogenous opioids that transit from the maternal blood to the placenta can thus impact this system. Effects of OXY and other opioids have been examined in cultured TB cells and shown to affect production of steroid hormones, HCG, and other placental factors (Cemerikic et al., 1988; Zharikova et al., 2007; Neradugomma et al., 2017; Serra et al., 2017).

We tested whether maternal OXY exposure affects the morphology and transcriptome profile as determined by RNAseq in E 12.5 mice placenta (Green et al., 2020). Maternal OXY treatment reduced pTGC area and maternal blood vessel area within the labyrinth region. OXY exposure altered placental gene expression profiles in a sexually dimorphic manner with female placenta exhibiting up-regulation of several placental enriched genes, including *Ceacam11*, *Ceacam14*, *Ceacam12*, *Ceacam13*, *Prl7b1*, *Prl2b1*, *Ctsq*, and *Tpbpa*. Placenta of OXY exposed males had alterations of many ribosomal proteins. Weighted correlation network analysis revealed that in OXY females vs. CTL females, select modules correlated with placental histological changes induced by OXY. Such associations were lacking in the male OXY vs. CTL male comparison. Pathways that are likely affected in OXY females based on gene-sets in these modules include extracellular matrix reorganization, VEGF signaling, and regulation of actin bioskeleton, collagen biosynthesis, peptide hormone signaling, interferon signaling, interferon gamma signaling, and triglyceride metabolism and catabolism.

### Smoking

Inorganic arsenic in cigarettes can affect placental architecture and function as discussed below. Other chemicals within cigarettes may also act though upon the placenta. The placenta expresses nicotinic acetylcholine receptor (nAChR) subunits that regulate TB cell invasion but whose expression and signaling pathway can be usurped by nicotine contained within tobacco smoke (Lips et al., 2005; Machaalani et al., 2014, 2018; Aishah et al., 2017; Chen et al., 2020). For instance, nicotine acting through nAChR induced endoplasmic reticulum stress in rat pTGC (Wong et al., 2016). Maternal smoking has been linked with changes in gross placental weight and microanatomical structure, especially for the extravillous TB cells (Heidari et al., 2018a,b; Larsen et al., 2018). Metabolism of lipids, namely long-chain polyunsaturated fatty acids and transporters for glucose uptake transporters (*SLC2A1* and *SLC2A3*), amino acids (*SLC7A8*), and lipid gene expression patterns in the placenta correlate with maternal smoking (Walker et al., 2019; Weinheimer et al., 2020). Circulating levels of placental-associated proteins, pregnancy-associated plasma protein A (PAPP-A) and free (fβHCG) are reduced in the serum of pregnant mothers who smoke (Jauniaux et al., 2013).

Transcriptomic and DNA methylation studies have been undertaken to determine whether maternal smoking affects these parameters in the placenta. An Illumina Expression Beadchip v3 that contained 24,526 transcripts was used to survey placental samples and cord blood from women who smoked while pregnant vs. non-smokers (Votavova et al., 2011). Pathways that were likely affected in placental and cord blood samples included xenobiotic metabolism, oxidative stress, inflammation, immunity, hematopoiesis, and vascularization. An earlier array-based study by this same research group found that maternal smoking induced several genes involved in xenobiotic metabolism (*CYP1A1, CYP1B1, CYB5A*, and *COX412*) collagen-associated genes (*COL6A3, COL1A1*, and *COL1A2*), coagulation genes (*F5* and *F13A1*), and thrombosis-related genes (*CD36*, *ADAMTS9*, and *GAS6*) (Bruchova et al., 2010). Another study that considered effects of maternal smoking on gene expression and the proteome in the placenta found that smoking down-regulated SERPINB2, FGA, and HBB but upregulated SERPINA1, EFHD1, and KRT8 (Huuskonen et al., 2016). Transcript expression for *CYP1A1* and *CYP4B1* were elevated, whereas *HSD17B2*, *NFKB*, and *TGFB1* were suppressed by maternal smoking (Huuskonen et al., 2016).

A handful of studies characterized DNA methylation changes in the placenta based on maternal smoking (Suter et al., 2011; Chhabra et al., 2014; Tsaprouni et al., 2014; Maccani and Maccani, 2015; Fa et al., 2016; Morales et al., 2016; Cardenas et al., 2019; Rousseaux et al., 2020). Exposure to maternal smoking during the first trimester increased methylation of the *AHRR* gene but did not alter its gene expression pattern (Fa et al., 2018). In contrast, maternal smoking during this period did not alter DNA methylation of *CYP1A1* but expression of this gene was upregulated (Fa et al., 2018). DNA methylation analyses with the Illumina HumanMethylation450 BeadChip for participants in the Infancia y Medio Ambiente (INMA) birth cohort revealed that maternal smoking decreased methylation levels of cg27402634 in the placenta, and this change was also associated with decreased birthweight (Morales et al., 2016). Another group that used this same BeadChip reported CpG sites mapping to *GTF2H2C* and *GTF2H2D* in the placental methylome strongly associated with maternal smoking (Chhabra et al., 2014). Usage of the Illumina HumanMethylation BeadChip technology in another cohort population showed that methylation patterns within the *RUNX3* gene were linked to maternal smoking during pregnancy with one of the loci correlating with decreased gestational age (Maccani et al., 2013).

One study considered whether maternal cessation of smoking prior to pregnancy would prevent some of the harmful DNA methylation marks relative to women who continued to smoke throughout their pregnancy (Rousseaux et al., 2020). The placenta from both groups of women showed similar epigenetic changes, including demethylation of LINE-1 sequences, enrichment in epigenetic marks for enhancer regions (H3K4me1 and H3K27ac), and regions in proximity or overlapping imprinted genes (*NNAT, SGCE, PEG10, H19.MIR675*). The persistence of DNA methylation changes in those women who quit smoking before becoming pregnant is worrying as it is suggests that events even during the periconception period can lead to a permanent stamp on the DNA methylome of the placenta. Further work is clearly needed to determine the extent to when such changes become irreversible and whether these same methylation alterations are conferred to the placenta of subsequent generations, i.e., potential transgenerational effects. In this aspect, another study reported that while cigarette smoking by pregnant mothers reduced DNA methylation for several genes, including *CPOX* near *GPR15, PRSS23, AVPR1B, PSEN2, LINC00299, RPS6KA2, and KIAA0087*, cessation of smoking 3 months prior to pregnancy partially reversed such methylation alterations in the placenta (Tsaprouni et al., 2014).

### Air Pollution

Particular matter that is around 2.5 μm in diameter (PM_2_._5_) in air pollution easily crosses the maternal-placental interface. As such, the placenta is vulnerable to such environmental toxicants. We will consider the evidence to date that air pollution disrupts the placental transcriptome and methylome profiles. Culturing of JEG-3 human placental cells in the presence of such PM affected genes involved in immune response, apoptosis regulation, calcium signaling pathway, steroid hormone biosynthesis, and cytokine-cytokine receptor interaction (Kim et al., 2018). Protein levels for mitogen activated protein kinases (MAPK) and COX2 were reduced in the PM_2_._5_ exposed JEG-3 cells. A Rhode Island Child Health Study (RICHS) revealed two developmentally sensitive windows to PM_2_._5_, with 12 weeks prior to and 13 weeks into gestation also being associated with reduced infant birthweight (Deyssenroth et al., 2021). This same study analyzed effects of PM_2_._5_ on placental gene expression patterns and relation to birthweight. Two placental modules enriched for genes involved in amino acid transport and cellular respiration correlated with maternal PM_2_._5_ exposure and infant birthweight (Deyssenroth et al., 2021). Additional findings from this cohort revealed that maternal exposure to PM_2_._5_ or black carbon (based on proximity to major roadways) changed the placental expression if several imprinted genes with *CHD7* showing interactions between PM_2_._5_ exposure/black carbon and infant sex being linked to placental expression of *ZDBF2* (Kingsley et al., 2017). The ENVironmental Influences ON early AGEing birth cohort was used to examine placental DNA methylation patterns (via a bisulfite -PCR pyrosequencing approach) in response to exposure to PM_2_._5_ or black carbon (Neven et al., 2018). Promoter methylation for *APEX1, ERCC4, and p53* were positively linked with maternal PM_2_._5_ exposure, whereas *DAPK1* showed a negative association to this extrinsic factor (Neven et al., 2018). Maternal exposure to black carbon was associated with increased promoter methylation for *APEX1* and *ERCC4*.

### MicroRNAs and Long Non-Coding RNAs

MicroRNAs (miR) and long non-coding (lnc)RNAs were once considered junk, but this “rubbish” RNA is now known to exhibit critical regulatory roles, including acting as the goalie as to which mRNAs are allowed to exist out of the nucleus and be translated to a protein vs. those that are instead targeted for degradation (Clancy et al., 2007; Van Wynsberghe et al., 2011; Moreno-Moya et al., 2014). Thus, increasing number of researchers are studying the expression of small and long non-coding RNAs in the placenta following maternal exposure to various xenobiotics. The expression pattern of such RNAs in the placenta might also provide key insights into infant diseases. For instance, several miRs, such as mR-379-3p, miR-335-3p, miR-4532, miR-519e-3p, miR-3065-5p, and miR-105-5p, were found to be down-regulated in the placenta of infants born small for gestational age (Östling et al., 2019).

BPA exposure has been linked to changes in miR expression patterns in whole placenta and TB cell lines (Avissar-Whiting et al., 2010; De Felice et al., 2015; Gao et al., 2018; Kaur et al., 2021). BPA treatment of human immortalized cytoTB cell lines and subsequent microarray analysis showed that several miRs had altered expression following this treatment, in particular miR-146a expression was strongly upregulated by BPA (Avissar-Whiting et al., 2010). Overexpression of miR-146a in these cell lines reduced cellular proliferation and rendered the cells more vulnerable to a DNA mutagenic agent (Avissar-Whiting et al., 2010). Genome-wide miR expression profiling revealed that maternal exposure to BPA significantly correlated with overexpression of miR-146a in whole placenta (De Felice et al., 2015). In experiments with California mice exposed to BPA, genistein, or the combination of these two EDCs, we found that developmental exposure to one or both compounds upregulated miR-146 in the hypothalamus of males and females (Kaur et al., 2021). In the testes, BPA induced miR-146a-5p that in turn impaired steroidogenesis through negative regulation of Metastatic tumor antigen 3 (MTA3) signaling (Gao et al., 2018). Taken together, miR-146 might be a biomarker for xenoestrogen exposure in mammals.

Analyses of miR expression patterns in placenta derived from a National Children’s Study (NCS) sought to link such profiles to maternal exposure to a variety of environmental toxicants, dichlorodiphenyldichloroethylene (DDE), bisphenol A (BPA), polybrominated diphenyl ethers (PBDEs), polychlorinated biphenyls (PCBs), arsenic (As), mercury (Hg), lead (Pb), and cadmium (Cd) (Li et al., 2015). PBDE 209 positively correlated with miR-188-5p but inversely associated with let-7c. PCBs and Cd positively associated with miR-1537 expression. Hg and Pb exposure were linked with down-regulation of several let-7 family members. However, maternal exposure to DDE or BPA levels were not associated with any changes in miR expression. The conflicting results between the one cohort study above that found a linkage between BPA and expression of miR-146a in the placenta and the NCS findings might relate to variation in BPA exposure for the cohort population of pregnant women examined, number of women enrolled, and sequencing technique, including depth of coverage.

The aforementioned Rhode Island Child Healthy Study explored the linkage between placental cadmium concentrations and lncRNA expression in the placenta (Hussey et al., 2020). MIR22HG and ERVH48-showed increasing expression corresponding to cadmium exposure and was linked with elevated odds of small for gesational age birth. In contrast, A114763.2 and LINC02595 demonstrated reduced expression relative to cadmium concentrations but increased odds for large for gestational age birth with increasing expression. In a Bangladesh cohort population with known exposure to arsenic, several placental miRs, miR-1290, miR-195, and miR27a, were negatively associated with birthweight, and miR-1290 expression varied based on arsenic exposure (Rahman et al., 2018).

One study explored whether maternal exposure to PM_2_._5_ air pollution during different pregnancy trimesters was linked with varying changes in placental miR (Tsamou et al., 2018). Accordingly, miR-21 and miR-222 expression in the placenta was inversely associated with PM_2_._5_ exposure during the 2nd trimester of pregnancy. However, exposure during the first trimester appeared to increase placental expression of miR-20a and miR-21. Based on the miR expression patterns, target mRNAs can be predicted, and the expression of tumor suppressor phosphatase and tensin homolog (*PTEN*) seems to be affected by the miRs that changed in response to maternal exposure to PM. Correspondingly, placental PTEN expression was positively associated with 3rd trimester PM_2_._5_ exposure (Tsamou et al., 2018).

## Conclusion

In acting as the gatekeeper, the placenta is vulnerable to xenobiotics circulating in the maternal bloodstream, especially in rodents and humans who exhibit an invasive, hemochorial type of placentation. In this review, we have considered the impact of EDCs, other environmental toxicants, pharmaceutical agents, maternal smoking, and air pollution on the placental transcriptome and other omics, including miR profiles, along with associated changes such compounds induced on placental morphology. What emerges from many of these studies is that such xenobiotics act upon endogenous receptors and transporters, e.g., steroid receptors, SERT inhibitors, opioid receptors, and nicotinic acetylcholine receptor, naturally expressed by the placenta that when bound by their endogenous ligands are crucial in regulating placental responses. Binding of xenobiotics to such receptors or transporters evades normally homeostatic regulation and prevents the endogenous ligands from binding and activating their cognate receptors.

**FIGURE 1:**
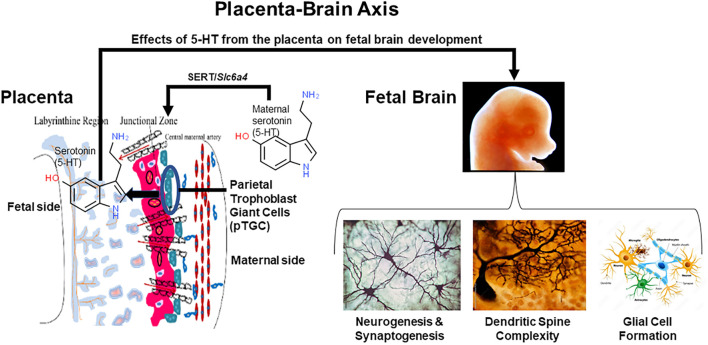
The placenta-brain axis and effects of placental 5-HT on fetal brain development. The parietal trophoblast giant cells (pTGC) in the fetal placenta may amass maternal 5-HT via SERT or may synthesize this morphogen. Placental-derived 5-HT may then act upon the developing fetal brain to promote neurogenesis, synaptogenesis, increase dendritic spine complexity, and glial cell formation. In essence, early embryology and brain development may depend upon 5-HT originating from the placenta.

The placenta is an ephemeral organ but how it responds to such xenobiotics can lead to longstanding effects on fetal health. Such is particularly true in the case of the fetal brain that depends upon placental hormones, especially 5-HT, for initial forebrain development (Bonnin and Levitt, 2011; Rosenfeld, 2020, 2021). The current studies show that exposure to BPA, VPA, and flame retardants suppress placental 5-HT, dopamine, and likely other neurotransmitters (Rubinchik-Stern et al., 2018; Mao et al., 2020; Rock et al., 2020). Such changes assumingly disrupt paracrine actions that these factors would otherwise stimulate in the placenta, but such placental disturbances can also lead to ramifications on the fetal brain whose initial development depends upon placental transfer of such substances, in particular placental derived 5-HT (Bonnin and Levitt, 2011; Rosenfeld, 2020, 2021). 5-HT acting as a morphogen may be one of the primary conductors regulating early neural crest formation, metamorphosis, neurogenesis, cell motility, synaptogenesis, and development of the nociceptive system (Lauder, 1988; Lauder et al., 1988; Whitaker-Azmitia, 1991; Herlenius and Lagercrantz, 2001). Definitive evidence that the placenta is the initial source of 5-HT for the developing fetal brain comes from studies that blocked placental tryptophan hydroxylase 1 (TPH1) enzymatic activity by *in utero* injecting the TPH inhibitor p-chlorophenyalanine (PCPA) into the labyrinth region of E14.5 placentas (Bonnin and Levitt, 2011). Predictably, direct and short exposure to this pharmacological inhibitor suppressed 5-HT levels in the placenta, as well as notably in the fetal forebrain. Thus, factors that affect placental synthesis and ability to amass 5-HT can also dramatically shape early brain development that can result in longstanding neurobehavioral changes. This inter-connection between the two organ systems, has been branded, the placenta-brain-axis (Rosenfeld, 2021). Placental transmission of 5-HT is likely one of many ways the placenta influences fetal brain development, as shown in Figure 1. Many neurobehavioral disorders likely trace their genesis to pathophysiological changes in the placenta (Marsit et al., 2012; Lesseur et al., 2014; Rosenfeld, 2015a).

One thing that also stands out in tracing the journey of discovery based on these omics approaches is that the experiments detailed herein employed techniques considered innovative for their time. However, ideas and technologies continue to evolve, and we must adapt our approaches accordingly. The studies described in this review were done with either whole placenta or isolated TB cell lines. Yet, the placenta in rodents and humans is a complex mixture of cells, and it is important to pinpoint how xenobiotics affect individual placental cell populations and how such changes may affect neighboring cells. The recently developed Visium spatial transcriptomics (ST) technology from 10X Genomics allows quantification of mRNA populations in the spatial context of intact tissue (Berglund et al., 2018; Maniatis et al., 2019). We have recently used this approach with mouse uteri (Mesa et al., 2021), but no studies to date have used novel method with placental samples. Single cell and single nuclei RNA-seq have been performed to understand human and mouse TB differentiation (Liu et al., 2018; Suryawanshi et al., 2018; Pique-Regi et al., 2019; West et al., 2019; Marsh and Blelloch, 2020). The architectural landscape of the placenta though is destroyed with both of these approaches. Thus, ST technology where the histoarchitecture is retained may complement these other techniques that allow for examination of gene expression patterns at the cellular or tissue level. Such approaches are predicted to become available to be able to characterize how varying extrinsic and intrinsic factors affect the DNA methylome, proteome, and global miR/other small RNA expression patterns down to the cellular level. MixOmics analyses (Rohart et al., 2017) and other integrative correlation analyses to be developed will permit integration of omics data and establish linkages between such molecular alterations to phenotypic changes in the placenta.

In conclusion, the studies to date provide strong evidence that xenobiotics affect placenta structure and molecular processes that assumingly affect placental morphology. The coming years will assuredly be devoted to tracing xenobiotic affects to individual TB cell populations and how such effects change over the course of pregnancy. How xenobiotics modify the placenta-brain axis is also predicted to become an important avenue of research. Identification of such placental changes may provide a mechanistic understanding of the fetal origin of neurobehavioral disorders in general and open up new avenues for early diagnosis and potential treatments for patients with autism spectrum disorders (ASD) caused by placental dysfunction during pregnancy.

## Author Contributions

CR researched, wrote, and edited the article.

## Conflict of Interest

The author declares that the research was conducted in the absence of any commercial or financial relationships that could be construed as a potential conflict of interest.

## Publisher’s Note

All claims expressed in this article are solely those of the authors and do not necessarily represent those of their affiliated organizations, or those of the publisher, the editors and the reviewers. Any product that may be evaluated in this article, or claim that may be made by its manufacturer, is not guaranteed or endorsed by the publisher.
